# mHealth and Engagement Concerning Persons With Chronic Somatic Health Conditions: Integrative Literature Review

**DOI:** 10.2196/14315

**Published:** 2020-07-24

**Authors:** Hanna Tuvesson, Sara Eriksén, Cecilia Fagerström

**Affiliations:** 1 Department of Health and Caring Sciences Linnaeus University Växjö Sweden; 2 Blekinge Institute of Technology Karlskrona Sweden; 3 Department of Health and Caring Sciences Linnaeus University Kalmar Sweden; 4 Blekinge Centre of Competence Blekinge County Council Karlskrona Sweden

**Keywords:** engagement, eHealth, mHealth, somatic disease, integrative literature review, telehealth

## Abstract

**Background:**

Chronic somatic health conditions are a global public health challenge. Being engaged in one’s own health management for such conditions is important, and mobile health (mHealth) solutions are often suggested as key to promoting engagement.

**Objective:**

The aim of this study was to review, critically appraise, and synthesize the available research regarding engagement through mHealth for persons with chronic somatic health conditions.

**Methods:**

An integrative literature review was conducted. The PubMed, CINAHL, and Inspec databases were used for literature searches. Quality assessment was done with the guidance of Critical Appraisal Skills Programme (CASP) checklists. We used a self-designed study protocol comprising 4 engagement aspects—cognitive, behavioral and emotional, interactional, and the usage of mHealth—as part of the synthesis and analysis.

**Results:**

A total of 44 articles met the inclusion criteria and were included in the analysis. mHealth usage was the most commonly occurring engagement aspect, behavioral and emotional aspects the second, cognitive aspects the third, and interactional aspects of engagement the least common aspect in the included articles. The results showed that there is a mix of enablers and barriers to engagement in relation to the 4 engagement aspects. The perceived meaningfulness and need for the solution and its content were important to create and maintain engagement. When perceived as meaningful, suitable, and usable, mHealth can support knowledge gain and learning, facilitate emotional and behavioral aspects such as a sense of confidence, and improve interactions and communications with health care professionals.

**Conclusions:**

mHealth solutions have the potential to support health care engagement for persons with chronic somatic conditions. More research is needed to further understand how, by which means, when, and among whom mHealth could further improve engagement for this population.

## Introduction

### Background

The growing burden of chronic health conditions has been described as a challenge and threat to health worldwide [1]. Ischemic heart disease, cerebrovascular disease, chronic respiratory conditions, and diabetes are the leading causes of death [2] and are predicted to continue increasing through the year 2030, according to a projection made in 2006 [3]. To address these challenges, patient engagement in treatment and care has been suggested as a key and powerful resource [4], with a shared understanding in the health care sector that engaged patients could improve quality of care and reduce unnecessary costs [5]. As part of ongoing developments in the field, health care organizations and national health care policies in many countries have recently begun focusing on engaging patients in the management of their health [6]. Various strategies and methods, such as electronic health (eHealth) and mobile health (mHealth), have been considered for facilitating and maintaining engagement with patients who have chronic somatic health conditions. Despite increased attention, however, there is still a lack of compiled information regarding engagement when using technological solutions as support in the care and management of health.

Although there seems to be a consensus concerning the importance of engagement in relation to living with and managing chronic health conditions, the concept of engagement in this context is underdefined, with several terms and definitions available to describe the phenomenon, and there is no shared understanding of how to conceptualize it [4,7]. Aside from *engagement*, commonly used terms are *commitment*, *activation*, *involvement*, and *adherence*. However, the concept of engagement conveys a somewhat divergent meaning from the other listed concepts, implying emotional commitment and involvement of the engaged individual. Moreover, depending on the discipline involved, the various preferences for which term to use and who should be engaged further complicate the understanding of the concept. Commonly used terms are *patient engagement* [4,8], *citizen engagement*, and *client engagement* [4]. *Consumer engagement* is another commonly used term [4,5], whereas in computer science, where the object and objective of engagement may often be more clearly delimited to a specific technological solution and its design process, *user engagement* is frequently used.

The American Health Information Management Association (AHIMA) [5] states that engagement comprises various activities such as interacting with health care professionals, seeking health information, maintaining a personal health record, and playing an active role in making decisions related to personal health care. Definitions of engagement in the literature have also been reviewed and categorized as *intraindividual factors* such as emotional, behavioral, and cognitive factors, and as a function of *interindividual factors* in terms of relationships and interactions. Many of the definitions have been suggested to be oversimplified and lacking in consideration of the progressive development of engagement over time as a process [4]. A recent review found that engagement in relation to digital behavior change interventions could be understood as 2 constructs: a subjective experience and behaviors [7]. Engagement in relation to digital and technological solutions has been described in terms of usage and patterns of usage of a product, such as the number of logs and time required to use the tool [9]. In this review engagement is understood as a multidimensional progressive process comprising cognitive, emotional, behavioral, relational, and interactional elements, as well as the usage of a product, service, or system.

Increasingly, various technological solutions are being suggested to support the health management of persons with chronic health conditions and to enhance their engagement. Mobile technology has, for example, been suggested to have an important role in facilitating patient engagement [10]. mHealth has been defined as the delivery of health care via the generation, aggregation, and dissemination of health information using mobile or wireless devices and the sharing of that information between patients and providers [5]. According to the World Health Organization [11], mHealth refers to medical and public health practices supported by mobile devices, such as mobile phones, patient monitoring devices, and personal digital assistants. In a systematic review [12], 3 distinct periods were identified with regard to the mobile devices used in mHealth research: before 2007, personal digital assistants dominated, while basic and feature phones took the lead during 2007-2012, after which smart devices came to dominate mHealth research. Over the past decade, there has been a considerable increase in the number and range of mHealth solutions that have been developed and implemented [11]. Because of their popularity, availability, portability, and technological capacity, smartphones and mHealth have enormous potential to impact chronic disease management around the globe [13]. However, so far, evaluations of mHealth solutions that focus on coverage, functionality, and impact on public health are few and far between [11]. In addition, in a systematic review of the impact of mHealth chronic disease management on treatment adherence and patient outcomes, Hamine et al. [13] argue that the impact of mHealth tools on adherence to treatment regimens may be overlooked because mHealth promoters are mainly focused on demonstrating their more direct effects on clinical outcomes (eg, morbidity, mortality, and biometric markers of clinical disease), while the long-term and more indirect effects of mHealth tools on adherence to treatment have not been in focus. Adherence to treatment, specifically adherence to treatment of chronic diseases, they argue, is critical to achieving improved health outcomes, quality of life, and cost-effective health care [13].

Various factors that are influential in the engagement process concerning mHealth solutions have been described; for example, in relation to the technological device/system (such as smartphone/computer access and technological shortcomings), the context (such as internet access and settings) and targeted behavior (such as expectations and meaningfulness) [5,7,10] are important factors. A known consumer/patient engagement challenge is that the patient may be uninterested in becoming engaged in the health care delivery process. Consumers are becoming more concerned with personal information security, including scenarios of hacking or identity theft, and in some cases, they have decided not to interact with online technology solutions. One of the major obstacles that providers face is that their patients’ health information is not always accurately and easily exchanged from one provider to the next [5]. The ability for patients and caregivers to access their health information electronically has been described as facilitating engagement [6], and maintaining engagement could improve personal and public health, patient experiences, and cost-reduction efforts [5]. Therefore, overcoming barriers to engagement and finding ways for those with chronic somatic health conditions to become engaged in their personal health and maintain that engagement are crucial. At this point, it is essential to review the research with regard to engagement through mHealth and to present the knowledge to identify the gaps and needs from the perspectives of those living with chronic somatic health conditions.

### Objectives

The aim of this study was to review, critically appraise, and synthesize the available research regarding engagement through mHealth for persons with chronic somatic health conditions.

## Methods

### Literature Search and Selection

An integrative literature review was made following the description of Whittemore and Knafl [14]. Systematic literature searches were performed in 2017 using 3 electronic bibliographic databases: CINAHL, PubMed, and Inspec. Two blocks of keywords were used to build the search strings for the identification of studies regarding engagement and mHealth among those with chronic somatic health problems: (1) engagement and (2) mHealth. Different forms (ie, inflections of engagement) of the word *engagement* were taken into account and used as keywords for the first block of the search. For the second one, (1) a list of keywords that were synonymous with or related to mHealth and (2) relevant MeSH and CINAHL headings were used as keywords, in combination. The concept of mHealth is embedded in the MeSH and CINAHL headings telemedicine and telehealth, and these were therefore used as keywords. A search string was conducted by combining the engagement keywords and the mHealth keywords with the Boolean operator OR. These 2 blocks of keywords were then combined with AND to complete the search string. The keywords and MeSH and CINAHL headings are presented in Textbox 1.

A total of 1907 references were found in the 3 databases. After the removal of duplicates, studies not written in English, and studies that were obviously irrelevant due to content or article type, 501 studies remained for further detailed screening. These 501 studies were screened using the abstract to determine eligibility. Studies were included if they (1) included patients 18 years of age or older, (2) were written in English, and (3) reported empirical results concerning engagement with patients suffering from chronic somatic health problems using, or participating in the design or research of, mHealth solutions. In the screening process, studies describing solutions that could be accessed through mobile or wireless devices were considered as mHealth [5]. No restriction by publication year was made as we anticipated that articles focusing on mHealth would be published in recent years. Studies were excluded if they focused on infectious and psychiatric conditions or reported on various injuries. Studies with an entirely preventive focus were also excluded. Several articles were excluded as they did not report any empirical findings; for example, editorials, letters, and study protocols. We were also unable to retrieve 7 of the references in full-text versions.

**Figure 1:**
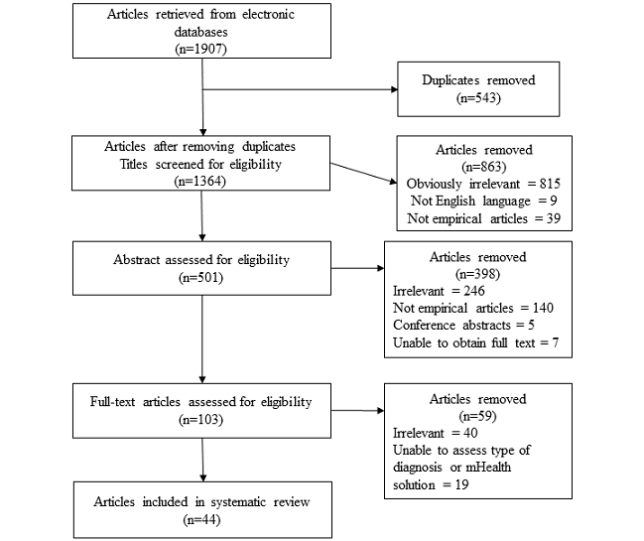
Flowchart of the data selection process.

In the last step of the screening process, the full-text copies of 103 articles were reviewed by 2 authors (HT and CF), and the results from the reviews were compared and discussed until there was acceptable inter-reliability between the reviewers (κ=0.9). Studies with mixed diagnoses were excluded if the results could not be directly related to one specific chronic health condition or if there was difficulty determining what type of technological solution had been used. Studies were also excluded if the results did not report findings on engagement in relation to the mHealth solution. In the end, we identified 44 studies as suitable according to the inclusion criteria. A detailed description of the data screening process is shown in Figure 1.

**Search strategy and used search terms.**

**Search terms**

- Engage OR Engaging OR Engaged OR Engagement AND Search terms (mHealth OR M health OR mobile health OR m-health OR mobile phone/-s OR mobile telephone/-s OR smart phone/-s OR cellular phone/-s OR cellular telephone/-s OR cell phone/-s OR cell telephone/-s OR telehealth OR Telemedicine)
- Engage OR Engaging OR Engaged OR Engagement AND MeSH and CINAHL headings (cell phones OR cellular phone OR smart phone OR smartphone OR telemedicine OR Telehealth)

### Quality Assessment

The 44 included studies were critically assessed for methodological quality using the CASP checklists for qualitative, randomized controlled trials, case–control, and cohort studies [15]. For the included mixed-methods studies, 2 checklists were used. The checklists, used worldwide, each comprise 10-12 questions with a numeric value representing each answer. All numeric values, based on subjective assessments, were summed to create a quality summary of each study. Because the quality assessment was part of the compilation, all studies screened for this integrative review were kept for further analysis regardless of the results of the quality assessment. A summary of the quality assessments for the 44 included studies is presented in Multimedia Appendix 1.

### Data Analysis

Data were identified and extracted from the articles based on protocols designed for this review by 2 authors (HT and CF; Multimedia Appendix 1). The protocol comprised information about the studies in terms of country, year of publication, study methods, type of mHealth solution, type of chronic somatic health condition, number of study participants, mean age of participants, duration of project or intervention, and whether information about research ethics was provided. Research ethical aspects, such as informed consent, voluntariness, and if applicable, advice or approval from ethical committees, is important when researching health aspects in patients and we decided to add this aspect in the study protocol to provide an overview. The protocol also comprised 4 aspects of engagement to be identified in the studies. These aspects were chosen based on definitions and descriptions in the literature of engagement as a process [4,5,7,9]. They were (1) cognitive aspects (eg, learning, understanding, knowledge), (2) behavioral and emotional aspects (eg, action, motivation, confidence, self-management, involvement), (3) interactional aspects (eg, relationships, communication with and accessibility to health professionals), and (4) mHealth usage (eg, participation, responses, logs, duration). In case of overlapping meanings with regard to the engagement aspects in the same article, data were sometimes reused in more than one engagement aspect. The results of the data identification completed by 2 reviewing authors (HT and CF), independently and according to the protocols, were compared and showed high levels of agreement. The results of the identification are detailed in the study protocol shown in Multimedia Appendix 1.

In the next stage of the analysis, data related to the 4 engagement aspects were identified and retrieved from the articles and then assembled in a document by one author (HT). The retrieved data were then read and compared with the aim of this study by all 3 authors. Even at this stage, ambiguous data were discussed until consensus was reached. The data were compared, and patterns and contrasts noted, before the analysis was discussed and revised until consensus was reached among the authors. Verification was conducted by comparing the results of the data analysis with the original articles.

## Results

### Description of Studies

Of the 44 articles included in this review, half (n=22) were published in the years 2015 and 2016. The majority of the studies were conducted in the United States (n=26), and the designs were mainly mixed-method or randomized controlled trials. Several studies compared groups, such as in quasi-experimental studies with a study group and a control group. Some studies (n=5) were based only on an analysis of program or system data, whereas the rest were qualitative studies, surveys, or case studies. The included studies used a mix of different mHealth solutions, including apps, interactive voice response, short message service (SMS), telemonitoring, websites, and personal health records. The duration of the study period varied greatly, from a couple of hours in a laboratory experiment to 2 years of follow-up. No information about research ethical approval or ethical aspects of the research was given in 10 of the 44 included studies.

Study participants were between ages 28 and 88, with an overall mean age of 57. The majority of the studies included individuals with diabetes or cardiac diseases or both (n=36). Several studies focused on hypertension or pulmonary diseases. There were also articles involving patients with kidney disease, pain, spina bifida, osteoarthritis, and cystic fibrosis. A total of 20 studies reached high methodological quality according to CASP, whereas 21 reached medium quality, and 3 studies had low quality (Multimedia Appendix 1).

### Engagement and mHealth

#### Cognitive Aspects of the Engagement Process

A total of 22 studies described cognitive aspects of the engagement process (Multimedia Appendix 1). Many of the studies reported that the mHealth solution supported engagement by providing persons with chronic health conditions with information about their health and their medical condition through personal health information, patient education in various forms, and a blend of feedback strategies [16-21]. This was, for example, described in ways such as seeking information on a website housed within a patient portal section of a public website [17] and gaining immediate and accurate information about one’s health status and health management, for example, by the tracking of blood glucose level, weight, and nutritional information based on food intake [20,21]. In some studies, feedback from the mHealth product resulted in disengagement if it was perceived as confusing, inaccurate, or complicated [22,23].

Persons with chronic health conditions learned new skills and gained knowledge about their health and medical conditions as a form of engagement when using mHealth solutions [16,17,19,20,24-31]. The learning process as a form of engagement was expressed in various ways. For example, mHealth solutions with coaching and educational components, such as animated scenarios, combined audio and text instructions and quizzes, supported learning [16,28,29].

Cognitive aspects of the engagement process were also described as understanding and awareness. Receiving personal, immediate, and understandable feedback about their clinical results and health management, based on self-entered information or clinical responses or both after health care visits, increased understanding and awareness [18,22,26,27,32-35]. Feedback such as SMS text messages or illustrative graphs helped patients understand and be aware of the relationships between clinical values, trends, and early warning signs of potential problems [26,34,35]. For some persons, the understanding and reflection first began after receiving glycemic readings that were outside of the recommended range or were unexpected [35].

#### Behavioral and Emotional Aspects of the Engagement Process

A total of 26 studies in this review included behavioral and emotional aspects as a form of engagement, often in relation to self-management and self-care components. Several studies reported that the mHealth solution supported management or improved self-management (Multimedia Appendix 1). For example, in one study, 64% (18/28) of the participants with diabetes type 2 thought that an iPad (distributed to them with installed existing apps) had helped them manage their diabetes [20], 97.4% (793/814) of patients with hypertension felt more confident in taking their blood pressure after using a telehealth programme [32], and patients in cardiac rehabilitation perceived a smartphone app to have supported their adherence to cardiac rehabilitation activities [36].

Several studies reported on significant improvements in self-management and self-care. For example, in one telemedicine study, people with diabetes type 2 had significantly improved self-efficacy after 2 years [37]. Significant improvements in self-care have also been found when comparing a telehealth group and a control group [38], and in another study, participants with osteoarthritis who had 12 months of exposure to a freely available mHealth website with evidence-based information and self-management resources had significantly improved self-management compared with nonusers [39]. In one study where both the study group and the control group comprised patients with chronic heart failure, both groups showed significant improvements in the self-management of their disease [40].

In several studies, SMS text messages or interactive voice response calls were found to be helpful with behavioral aspects of engagement [22,33,41,42]. Some users perceived reminders and alarms as helpful for engaging in health-related activities [19,29], but they could also be seen as disengaging by being nagging [19]. Automatic feedback messages or system feedback has been used to support behavioral and emotional aspects of engagement. For many people, these messages were perceived as beneficial in that system feedback reinforced positive changes in behavior and helped break negative cycles [43], helped patients establish and achieve goals [25,35], helped to establish routines [33], and reinforced compliance [36]. In at least one study, online stand-alone personal electronic health records were found to be not crucial for daily self-management [18].

Different mHealth solutions, such as telemonitoring and various apps, supported emotional components of the engagement process. For example, solutions were found to support or enhance motivation [19,23,29,31,43], provide a sense of control and mastery [20,23,27,29], boost confidence [20,21,29,35,40], and give users a sense of empowerment [20,21,26] through self-care and management of their illness.

Conversely, some mHealth solutions also created emotional hindrances to engagement. The tools could lead to less self-management after the novelty phase compared with that at the start of the program [20], and in some cases, users would experience worry and depressive feelings when readings were worse than normal, leading to a decline in self-management [27]. Still, other patients did not want to use and interpret readings, preferring to leave that to health professionals [27]. Further, patients who were already motivated before using an app or other tool did not necessarily become more motivated [31].

#### Interactional Aspects of the Engagement Process

The 18 studies describing interactional aspects of the engagement process were primarily focused on ways of communicating and sharing information, such as with regard to physical visits to health care professionals (Multimedia Appendix 1). The experiences of users included feelings of being continuously connected to health care professionals [26,27,33,36,44,45] and having increased access to care [26,46]. Some users thought that the mHealth solution had improved their visits to health care professionals [36], and a few studies found that the mHealth solution (including small-sized peripheral telehealth equipment with individual reading and teletherapy technology with tailored counseling) prevented admission to hospitals [26,47]. However, another study showed that hospital visits had increased in both the control group and the intervention group [48].

In one study, shared information was seen by some patients as beneficial for engagement, but most participants did not share their personal electronic health record with health care professionals [18]. Knowing that health care professionals were able to access their electronic health records and see recent test results or monitor readings made many users feel safe, peaceful, and watched over [26,27,49]. However, others felt uncomfortable or unsafe with their personal health information being shared in an mHealth system, feeling like they were under surveillance [29,49].

Many thought that communication through mHealth systems was insufficient in many ways, pointing to these tools’ lack of face-to-face contact or a sense of *human touch* [21,26], while others were concerned about the quality of the communication due to, for example, the lack of access to body language or physical demonstrations with equipment [26,48], which were perceived as creating disengagement.

#### mHealth Usage

A majority of the included studies (37/44) described the engagement process in relation to usage of the mHealth solution through process measurements, for instance, where engagement was reduced due to the high number of activities involved, such as completion of messages, log-ins required, and system tracking of various sorts (Multimedia Appendix 1). High mHealth utilization rates were reported in a number of studies [21,22,35,36,38,44,45,47,48,50], but a few described limited utilization [17,24,41]. In several studies engagement was related to the time spent using the mHealth solution and if or when participants dropped out or declined to use the solution. The analyses show a great variation in engagement as described in these papers. In several studies, a decline in usage was reported, irrespective of whether the studies had shorter or longer durations [23,32,45,46,50,51]. There were some studies that found that the majority of participants stayed interested and continued to use the mHealth solution throughout the study period [36,44,47,48].

Some studies found that engagement in terms of usage varied depending on the user and his or her situation. Older patients with poor health, for example, were found to be less engaged in various mHealth solutions [42,52]. At the same time, having a need that could be satisfied with an mHealth solution, such as a need for self-management assistance, led some people to maintain engagement [23,53], whereas in another case, a ceiling effect was found when blood glucose levels were under control [46]. One study found that unmarried persons had lower engagement [52] rates, and in several studies, participants with informal caregivers had high engagement rates [42,50,52,53].

Several factors were described as influencing the engagement process. Lack of time and busy lifestyles, with constraints due to home obligations, childcare, work, and travel leading to difficulties in integrating the solution into daily life, often resulted in disengagement [18,23,28,34]. Technological problems such as glitches, errors, connectivity problems, and battery attrition were other factors described as influencing engagement [23,28,31,34]. Concerns about trust, privacy, and security were also reported to influence engagement and usage negatively [18,19,34,43]. Conversely, visually exciting and dynamic components in the mHealth solution were factors that could stimulate engagement [19].

## Discussion

### Principal Findings

This study aimed at reviewing, critically appraising, and then synthesizing the body of research regarding engagement with mHealth solutions by persons with chronic somatic health conditions. In total, we found 44 studies that met the inclusion criteria and described aspects of the engagement process in terms of cognitive, behavioral and emotional, or interactional aspects, along with those regarding the specific usage of an mHealth solution.

### mHealth and the Engagement Process

Overall, the results of this review support the idea that engagement is a process and that cognitive, behavioral and emotional, interactional, and mHealth usage–related aspects of engagement go hand-in-hand and influence each other in many cases. The individual person and his or her situation, along with the type of health problem and the stage of some conditions, are important factors in mHealth use. The specific mHealth solution is also important in terms of content, usability, and visibility. When an mHealth solution is perceived as supportive and meaningful, the person’s knowledge and understanding of his/her health and interaction with health care services can be facilitated. This, in turn, may result in more awareness of self-management of health as well as emotional empowerment in terms of confidence and control. Previous studies have found that when data transmissions and health information are being transferred to health care professionals and information about the data is asynchronously being sent back to the patient, the patient sometimes feels more involved and engaged in the interpretations of data [54,55]. Yet, this could also lead to passivity and disengagement as the person’s personal interpretations of his or her health situation are being unattended in one-way communications [56]. mHealth solutions with self-care components convey an enhanced responsibility to someone with somatic health conditions, and therefore, these solutions need to be flexible when it comes to user preferences [54].

One interpretation of the results is that patient needs is one factor that could, alone, be a deal breaker for engagement. If the disease is under control, no new medicines have been added, or no new knowledge is needed, the engagement process may no longer be progressive in nature. The mHealth solution needs to serve an actual perceived need for the patient to maintain engagement with it. This was illustrated, for example, in the results with several studies that reported satisfying usage and engagement in the mHealth solution for the majority of the participants, but there were difficulties in maintaining engagement in the long term. This could possibly be related to ceiling effects and lack of perceived need to use the mHealth solution when knowledge gaps have been filled or when the disease is under control, and in this regard, a lack of engagement could be interpreted as something positive. These findings are in line with previous research where individuals ceased to use mHealth solutions when they had reached their goals, felt a lack of stimulation, or *outgrew* the solution [56], among other things. However, this insight could be used constructively when planning for mHealth interventions by targeting user groups in a more focused way, with the aim of reaching specifically those groups of people who are in a situation where the need for mHealth solutions is perceived as most pressing. Such *windows of opportunity* for achieving a high level of mHealth engagement could, for instance, be during the first 6 months after a person has been diagnosed with diabetes type 2, when the shock of the new situation, a lack of established habits and routines for dealing with it, and the need for information and deeper knowledge about the disease and how to manage it create a clearly perceived need for efficient mHealth support [57]. In addition, Zrebiec [58] found that people who have had diabetes for more than 2 years might have a need for information due to progression of the disease or the onset of diabetes-related complications or both. The literature suggests various options for maintaining engagement over time in mHealth solutions, such as with incentives. In a systematic review of incentive-driven mHealth solutions, the use of education, reminders, feedback, social aspects, alerts, gamification, and financial incentives were mechanisms found to engage and motivate users. The authors of that review discuss that although technological innovations change and develop, the incentives do not, and few new approaches have been presented [59]. The results of our review indicate that creative and intriguing mechanisms, such as artificial intelligence components (eg, customized SMS text message reminders, monitors, and avatars), could stimulate engagement. Furthermore, Liu and colleagues [60] found that loyalty rewards significantly influenced enrollment, but ongoing engagement was not influenced by the rewards. Stopping the usage of mHealth (nonusage attrition) or being lost to follow-up (dropout attrition) in mHealth research could be understood as the phenomenon of *the law of attrition* as formulated by Eysenbach [61]. According to Eysenbach, an innovation will be rejected if it is perceived as not providing any benefits or if there are usability problems. However, other factors are also important for understanding the engagement or disengagement process, such as demographics and study settings [62]. Furthermore, our review found that the context, characteristics of the person, and his or her life situation are crucial factors for engagement that need to be considered.

Of the 4 different aspects of engagement in mHealth among persons with chronic somatic health conditions as identified in this study, mHealth usage was the most commonly occurring aspect, identified in 37 of the 44 papers. The category of behavioral and emotional aspects was the second most common, identified in 26 of the 44. Cognitive aspects were identified in 22 articles, whereas interactional aspects of engagement were only identified in 18 articles included. A logical conclusion concerning this difference is that it indicates something about the history of research on engagement in mHealth; during the early years of mHealth studies, the focus would have primarily been on who was adopting the new technologies and what their associated characteristics (eg, demographic) were. Our hypothesis is thus that mHealth usage was the primary point of interest in the early years of mHealth studies and that studies of technology use have been historically heavily influenced by behaviorism and cognitive psychology. This could explain why behavioral and emotional aspects are the second most common aspects of mHealth engagement, as identified in the results of our study. Cognitive aspects come in third place, while interactional aspects come last of all, which is a bit surprising given that mobile technology has been developed primarily as a tool for communication and knowledge sharing. As such, it should be seen as supporting more efficient communication and interaction in health care. Why do we see relatively little focus on the interactional aspect of engagement in our study? Could this have historical roots in the health and medical sciences as well as in mHealth research, that is, could it be a reflection of a history heavily influenced by behaviorism and cognitive psychology and only more recently becoming influenced by social psychology, phenomenology, and social anthropology as well as inspired by work practice theory, ethnographic and ethnomethodological studies, and participatory design? Such an interpretation resonates with recent and current discourse in human–computer interaction research on a perceived ongoing transition between second- and third-generation theory and technology [62,63], a transition characterized by a shift of focus from *human factors* to *human actors*, and a more human-centered and participatory approach to the design and use of technology [64].

It is also interesting in a study such as this to explore what might have been expected but appears to be missing in the results. Most of the articles included in this literature review related to diabetes and heart disease, concerned older patients, and were conducted in middle- and high-income countries. This might mean that the engagement process in youths or young adults, in patients with other chronic somatic health conditions, or in developing countries will involve different enablers and barriers that are not visible in this review. Thus, there appears to be a need for more research into mHealth tools as they relate to other chronic somatic health conditions, different populations, and across wider geographical areas.

### Design and Quality of the Studies

The included studies varied in methodological design, which imposed a few challenges in terms of quality appraisal. For example, the methodological descriptions were sometimes limited due to mixed-method designs or for those involving larger ongoing projects where methodological information regarding the project had previously been published. Quality appraisal is a key component of a systematic review [65,66], but in mixed-method reviews, it can be difficult to find appropriate quality assessment guidelines that work for all included studies. Using mixed methods to understand complex phenomena and telehealth interventions is useful [67,68], yet threats to quality could be more difficult to identify compared with monomethod studies [68]. Because many of the studies in this review used a mix of methods in the same article, 2 CASP checklists were used for the same study in those cases.

No ethical aspects of research were mentioned in 10 of the 44 included studies. Arguably, research ethics must be considered the cornerstone of all research involving humans. In this review, we included research on persons with chronic somatic health conditions, a group that may be vulnerable or dependent on health care services. We realize that research ethics, laws, and standards vary among countries, but descriptions of particular aspects, such as protection from research-related harms, confidentiality, and informed consent, are important and applicable globally.

### Strengths and Limitations

Strengths of this review include that each stage of the study, such as the identification of keywords, applicable articles, quality assessment, and analysis, was conducted by more than one author with regular discussions held between authors to ensure consistency. The authors of this review have a mix of methodological experiences, which was a strength when reviewing and synthesizing the data. At the start of this study, the initial idea for the literature search was to use synonymous words for the concept of engagement, for example, *commitment* and *involvement*. When using these synonyms in the first search strings, however, it became apparent that the majority of the studies did not involve what we were setting out to review in terms of engagement. Other related words such as *patient/consumer facing* and *motivation* were also excluded from use, owing to the same reasons. We therefore decided to limit the use of search terms to inflections of the word engagement and to create a protocol for engagement aspects that could guide the review and analysis process based on the description of the concept guiding this review. The concept and phenomenon of engagement are complex, and there may be a risk that relevant data were missed due to this approach, but we believe that it was primarily a strength in relation to the aim of this review.

In the literature search, we used a broad definition of mHealth and included studies that we understood were using mobile or wireless devices [5]. The use of MeSH and CINAHL headings, such as telemedicine and telehealth, resulted in a screening process in which mobile or wireless components needed to be identified in order to pass as mHealth. This process led to the exclusion of articles in case there was an uncertainty regarding the telehealth approach and may have led to potential missed data. The use of MeSH and CINAHL headings is, however, an advantage in literature reviews that we wanted to use.

The majority of engagement results related to aspects of usage of mHealth solutions. Several of these results were difficult to analyze and synthesize due to difficulties in conveying the actual meaning of the results, such as what a certain number of log-ins could imply in relation to engagement. Without knowing if this quantity was as expected or not, a reliable interpretation is difficult to draw without risk of oversimplifying or overestimating the results. Therefore, we decided to only synthesize results in studies where the meaning was clear. This may have influenced the results and led to the exclusion of some findings, but it was a necessary approach to create trustworthiness in the analysis process.

The included quantitative studies did not yield aggregated data for use in a meta-analysis and thus data were synthesized in a more narrative way together with those from other studies, which was considered a suitable alternative. We used 3 databases for the search of studies. These were chosen because they were deemed to be the largest and most relevant databases for this review, although we retrieved few appropriate studies with the use of the Inspec database, which was somewhat surprising. It is possible that the choice of these 3 databases may have limited the number of included studies.

### Conclusion

Our integrative literature review revealed that engagement is a progressive process that comprises cognitive, behavioral and emotional, interactional, and mHealth usage–related aspects. To create and maintain sustainable engagement processes in mHealth research, enablers and barriers in all the engagement aspects might need to be acknowledged. mHealth solutions have the potential to support engagement in persons with chronic somatic health conditions when perceived as meaningful, suitable, and useful, but the research on engagement in relation to the implementation and usage of such solutions is mixed. Further research is needed concerning other chronic somatic health problems besides diabetes and heart disease. In addition, research focusing on finding new and innovative incentive mechanisms to support engagement when using mHealth solutions for persons with chronic health conditions could improve mHealth solutions to better support engagement. Sometimes, disengagement in terms of nonusage attrition could be a positive aspect, and it might be important to consider when and where to stake efforts when striving for long-term engagement.

## Appendix

Multimedia Appendix 1 Search protocol.

## Abbreviations

AHIMA: American Health Information Management Association
CASP: Critical Appraisal Skills Programme
SMS: short message service
