# Supplementary material for: mHealth and Engagement Concerning Persons With Chronic Somatic Health Conditions: Integrative Literature Review
Source: JMIR Mhealth Uhealth. 2020 Jul 24;8(7):e14315. doi: 10.2196/14315 (PMC7414402; doi:10.2196/14315)
Supplement: Multimedia Appendix 1 [file mhealth_v8i7e14315_app1.docx]

**Table 2.** Study protocol, summary of included studies, and quality appraisal.

| **Authors**  **Year**  **Country** | **Study type**  **Type of mHeath solution** | **Health condition**  **Sample size**  **Age**  **Duration of study**  **Ethical approval/consideration** | **Cognitive aspects** | **Behavioural and emotional aspects** | **Interactional aspects** | **mHealth usage** | **Quality appraisal^1^** |
| --- | --- | --- | --- | --- | --- | --- | --- |
| Agboola S, Havasy R, Myint-U K, Kvedar J, Jethwani K  2013  USA | Retrospective review of remote monitoring data with one wireless-based group and one modem-based group.  Data transfer technology. | Diabetes, hypertension  N=30  23-84 years old  Mean age=61.7 years  At least 2 month study duration  No information about research ethics |  |  |  | X | Medium |
| Aikens JE, Zivin K, Trivedi R, Piette JD  2014  USA | Observational study. Questionnaires by phone.  Mhealth IVR calls. | Diabetes type 2  N=303  Majority >60 years old  3-6 month study duration  Research ethical approval |  |  |  | X | High |
| Athilingam P, Osorio RE, Kaplan H, Oliver D, O’Neachtain T, Rogal PJ  2016  USA | Questionnaire with open and closed questions.  Beta testing of mobile application. | Heart failure  N=10  43-81 years old  Mean age=63  2-hour test  Research ethical approval | X |  |  |  | Medium |
| Brennan PF, Casper G, Kossman S, Burke L  2007  USA | Field experiment and case study in ongoing project. Usual care vs. technology-based care.  Web-based resource. | Chronic cardiac disease  N=24 (only those in the experimental group) + 2 patients’ case summaries  43-88 years old  6 month study duration  No information about research ethics | X |  |  | X | Low |
| Capozza K, Woolsey S, Georgsson M, Black J, Bello N, Lence C, Oostema S,North C  2015  USA | RCT with two arms, surveys, and data from text messaging programme.  Text messaging programme. | Diabetes type 2  N=93  Inclusion >18 years; no specific information about age  6 month study duration  Research ethical approval | X |  |  | X | Medium |
| Chen L, Chuang L‑M, Chang C‑H, Wang C‑S, Wang I‑C, Chung Y, Peng H‑Y, Chen H‑C, Hsu Y‑L, Lin Y‑S, Chen H‑J, Chang T‑C, Jiang Y‑D, Lee H‑C, Tan C‑T, Chang H‑L, Lai F  2013  Taiwan | Test and control group; system analysis.  Telehealthcare programme and online application, 3G glucometer, and teleconsultant service. | Diabetes types 1 and 2 with less controlled diabetes  N=59 (162)  Mean age=51.34 years  18 month study duration  Research ethical approval | X | X |  | X | High |
| Cook DJ, Manning DM, Holland DE, Prinsen SK, Rudzik SD, Roger VL, Deschamps C  2013  USA | Analysis of electronic health record data and programme data transmissions.  Electronic health information platform. | Cardiac disease, surgical, hospitalized  N=149  52-85 years old  Mean age=68  5-7 day study duration  Research ethical approval |  |  |  | X | Medium |
| Cottrell E, Cox T, O’Connell P, Chambers R  2015  UK | Automatic data processing from telehealth programme.  Telehealth with interactive messaging. | Hypertension  N=2963  No information regarding age  12 week study duration  Described as not being a research project and therefore in no need of research ethical approval | X | X | X | X | Medium |
| Duggan GB, Keogh E, Mountain GA, McCullagh P, Leake J, Eccleston C  2015  UK | Mixed methods: interviews and questionnaires.  Self-management system through touchscreen computer and mobile telephone. | Chronic pain  N=8  33-75 years old  Mean age=50.3  4 week study duration  Research ethical approval |  | X |  | X | Medium |
| Evangelista LS, Jung-Ah L, Moore AA, Motie M, Ghasemzadeh M, Mangione CM  2015  USA | Quasi-experimental study with a study group and a control group.  Remote monitoring system and telehealth communication. | Chronic heart failure  N=21  58-83 years old  Mean age=72.7  3 month study duration  Research ethical approval  . |  | X |  |  | High |
| Fioravanti A, Fico G, Salvi D, García-Betances RI, Arredondo MT  2015  Spain | RCT pilot with survey and log files.  mHealth application with automatic feedback messages. | Diabetes types 1 and 2  N=51 (I=26 + C=25)  No information regarding age  4 week study duration  No information about research ethics | X | X |  | X | Medium |
| Fischer HH, Moore SL, Ginosar D, Davidson AJ, Rice-Peterson CM, Durfee MJ, MacKenzie TD, Estacio RO, Steele AW  2012  USA | Quasi-experimental pilot study. Focus groups and analysis of logs.  Software platform with automatic text messages and processing of message responses. | Diabetes  N=47 (eight also participated in two focus groups)  40-70+ years old  3 month study duration  Research ethical approval | X | X | X | X | Medium |
| Forman DE, LaFond K, Panch T, Allsup K, Manning K, Sattlemair J  2014  USA | Observational pilot study with application reports.  Smartphone application with feedback messages. | Cardiac disease  N=26  43-76 years old  Mean age=59  30 day study duration  Research ethical approval |  | X | X | X | Medium |
| Fuji KT, Abbott AA, Galt KA  2015  USA | Qualitative study with interviews.  Electronic stand-alone personal health record. | Diabetes type 2  N=59  28-80 years old  Mean age=59  3-6 month study duration  Research ethical approval | X | X | X | X | High |
| Gellis ZD, Kenaley B, McGinty J, Bardelli E, Davitt J, Ten Have T  2012  USA | RCT with questionnaires and telehealth data.  Home Telehealth with telehealth monitoring and integrated electronic medical record. | Heart or chronic respiratory failure  N=115 (I=57 + C=58)  Mean age=79  12 month study duration  Research ethical approval |  |  | X | X | High |
| Gorst SL, Coates E, Armitage CJ  2016  UK | Qualitative study with semi-structured interviews.  Home telehealth and vital signs monitoring. | COPD  N=8  58-84 years old  Mean age=68  No information about research ethical approval; informed consent was applied | X | X | X |  | High |
| Hanley J, Fairbrother P, Krishan A, McCloughan L, Paterson M, Pinnock H, Sheikh A, Sudlow C, Todd A, McKinstry B  2015  UK | Pilot trial with mixed methods in two phases: 1) RCT pilot, and 2) interviews.  Telemonitoring, home blood pressure monitoring. | Stroke or transient ischaemic attack  N=55 (I=40+C=15)  16 interviews + 23 focus groups  Mean age=67.8 + 66 + 68  6 month study duration  Research ethical approval |  | X | X | X | High |
| Hilliard ME, Hahn A, Ridge AK, Eakin MN, Riekert KA  2014  USA | Mixed methods: web survey and semi-structured interviews.  mHealth application. | Cystic fibrosis  N=16  21-43 years old  Mean age=30.2l  Data on one occasion  Research ethical approval | X | X | X | X | High |
| Hong MK, Cho YY, Rha MY, Kim JH, Lee MK  2015  Korea | Single case study.  Mobile phone application, one session of telephone consulting. | Diabetes type 2  N=1  Age=46  6 month study duration  No information about research ethics |  | X |  | X | Low |
| Huniche L, Dinesen B, Nielsen C, Grann O, Toft E  2013  Denmark | Qualitative study with semi-structured interviews.  Self-monitored readings through telehealth monitoring. | COPD  N=22  Mean age=69.4 in males, 66.4 in females  16 week study duration  Research ethical approval | X | X | X |  | Medium |
| Koopman RJ, Johanning JL, Kruse RL, Bernt B, Mehr DR  2014  USA | Qualitative study in parallel with an RCT. Grounded theory based on notes from telephone exit interviews.  Home telemonitoring of blood glucose and blood pressure. | Diabetes  N=93  No information about specific age; only adults were included  3 month study duration  Research ethical approval | X |  |  | X | Medium |
| Koufopoulos JT, Conner MT, Gardner PH, Kellar I  2016  UK | Two-arm RCT with questionnaires and site activity data.  Web-based and mobile intervention with online community. | Asthma  N=216 (online community group=99; control group/diary only group=117)  18-64 years old  Mean age=28.1  9 week intervention  Research ethical approval |  |  |  | X | High |
| Mamykina L, Heitkemper EM, Smaldone EM, Kukafka R, Cole-Lewis H, Davidson PG, Mynatt ED, Tobin JN, Cassells A, Goodman C, Hripcsak G  2016  USA | Qualitative study with interviews and descriptive statistics of usage logs. Participants from an RCT.  Web-based application with integrated SMS capabilities and blood glucose readings. | Diabetes type 2  N=15  25-63 years old  Mean age=53  4 week study duration  No information about research ethics | X | X |  | X | High |
| Nelson LA, Mayberry LS, Wallston K, Kripalani S, Bergner EM, Osborn CY  2016  USA | Mixed methods: phone interview, surveys, and system responses.  Tailored short message service. | Type 2 diabetes  N=36  Mean age=52.4  2 week study duration  Research ethical approval | X | X |  | X | Medium |
| Nijland N, van Gemert-Pijnen JE, Kelders SM, Brandenburg BJ, Seydel ER  2011  Netherlands | Mixed methods: based on log files, e-mail interviews, tests, and surveys.  Web-based application with online health monitoring. | Diabetes type 2  N=50 (log files)  43-80 years old  Mean age=61  2 year study duration  No information about research ethics |  | X | X | X | High |
| Nundy S, Dick JJ, Chou C-H, Nocon RS, Chin MH, Peek ME  2014  USA | Quasi-experimental pre- and post-study based on questionnaires, response analysis, and HbA1c.  mHealth program with automated text messages. | Diabetes types 1 and 2  N=67  22-69 years old  Mean age=53  6 month study duration  Research ethical approval |  | X | X | X | Medium |
| Ong SW, Jassal SV, Miller JA, Porter EC, Cafazzo JA, Seto E, Thorpe KE, Logan AG  2016  Canada | Statistical analysis of application use and activity and semi-structured exit interviews.  Smartphone application with home blood pressure monitoring, tracking of laboratory test results, and personalized messages. | CKD  N=45  Mean age=59  6 month study duration  Research ethical approval |  |  | X | X | High |
| Park S, Burford S, Nolan C, Hanlen L 2016  Australia | Two online surveys (after 2 and 5 months). Open-ended and closed-ended questions.  mHealth program with built-in digital training support. | Diabetes type 2  N=28  30-79 years (82% were 50 years or older)  10 month study duration (data from 2 and 5 months)  Research ethical approval | X | X |  | X | High |
| Pekmezaris R, Schwartz RM, Taylor TN, DiMarzio P, Nouryan CN, Murray L, McKenzie G, Aern D, Castillo S, Pecinka K, Bauer L, Orona T, Makaryus AN  2016  USA | Focus group discussion in a randomized clinical trial. Community-based participatory research.  Telemonitoring system with interactive video monitoring system. | Heart failure  N=4  No information regarding age  3 month study duration  No information about research ethics | X |  |  | X | Medium |
| Pfaeffli L, Maddison R, Whittaker R, Stewart R, Kerr A, Jiang Y, Kira G, Carter K, Dalleck L  2012  New Zealand | Mixed methods: focus groups, individual telephone interviews, survey.  mHealth intervention, with short message service, website, and video vignettes. | Cardiac disease  N=38 in focus groups + 20 individual interviews  No specific information regarding age; 71% of participants were 56 years or older  No specific information regarding study duration  Research ethical approval | X |  | X | X | Medium |
| Piette JD, Rosland AM, Marinec NS, Striplin D, Bernstein SJ, Silveira MJ  2013  USA | Statistical analyses on IVR-reported data.  IVR calls. | Heart failure, diabetes, cancer  Heart failure: N=394, 12 month study duration  Diabetes: N=285, 12 week study duration  Cancer: N=52, 10 week study duration  Mean age=61  Research ethical approval |  |  |  | X | High |
| Piette JD, Marinec N, Gallegos-Cabriales EC, Mercedes Gutierrez-Valverde J, Rodriguez-Saldãna J, Mendoz-Alevares M, Silveira MJ  2013  USA, Mexico, Honduras | Statistical analyses on IVR-reported data. Honduras: pilot feasibility study. Mexico: pilot randomized trial. Honduras and Mexico: RCT. USA: pilot study.  IVR calls. | Diabetes or hypertension  N=268  Mean age=55  6 or 12 week study duration  Research ethical approval in each country |  | X |  | X | High |
| Piette JD, Aikens J, Rosland AM, Sussman JB  2014  USA | Statistical analysis of IVR-reported longitudinal data and baseline telephone surveys.  Interactive voice response calls. | Diabetes type 2, hypertension  N=298  No specific information regarding age; 82% of the participants were at least 60 years old  3 and 6 month study duration  Research ethical approval |  |  |  | X | High |
| Piette JD, Marinec N, Janda K, Morgan E, Schantz K, Aruquipa Yujra AC, Pinto B, Huayta Soto JM, Janevic M, Aikens JE  2016  Bolivia, USA | Randomized trial—with or without informal caregiver. Statistical analysis of baseline interviews and survey and IVR-reported data.  IVR calls. | Diabetes and/or hypertension  N=72  No specific information regarding age; 62.5% were 60 years or older  4 month study duration  Research ethical approval in both countries |  |  |  | X | Medium |
| Pludwinski S, Ahmad F, Wayne N, Ritvo P  2016  Canada | Qualitative study with semi-structured interviews alongside RCT.  Smartphone and self-monitoring software. | Diabetes type 2  N=11  Mean age=63.5  6 month study duration  Research ethical approval | X | X | X |  | Medium |
| Shane-McWhorter L, Lenert L, Petersen M, Woolsey S, McAdam-Marx C, Coursey JM, Whittaker TC, Hyer C, Lamarche D, Carroll P, Chuy L  2014  USA | Nonrandomized prospective observational pre- and post-intervention study. Statistical analyses of telemonitoring data and questionnaires.  Two telemonitoring delivery methods. | Uncontrolled diabetes with or without hypertension.  N=109  Mean age 50.6  6 month study duration  Research ethical approval | X |  |  | X | High |
| Tatara N, Årsand E, Skrøvseth SO, Hartvigsen G  2013  Norway | Mixed methods: questionnaires and interviews. Analysis of recorded data, usage trends, and patterns.  Mobile application, including automatic data transmission. | Diabetes type 2  N=12  44–70 years old  Mean age=55.1  1 year study duration  Research ethical approval | X | X | X | X | Medium |
| Tatara N, Årsand E, Bratteteig T, Hartvigsen G  2013  Norway | Mixed methods: trial with, questionnaires, focus group sessions, analysis of recorded data.  Mobile application, including automatic data transmission. | Diabetes type 2  N=11  40-73 years old  Mean age=57.2  5 month study duration  Approved by privacy officer at hospital; ethical approval was deemed unnecessary by the ethics committee | X | X |  | X | Medium |
| Triantafyllidis A, Velardo C, Chantler T, Ahmar Shah S, Paton C, Khorshidi R, Tarassenko L, Rahimi K  2015  UK | Mixed methods: observations, interviews, and review of physiological measurements and system usage logs.  Mobile-based home monitoring system, application, and sensing devices. | Heart failure  N=26  Mean age=72  1 year study duration  Research ethical approval |  |  |  | X | Low |
| Trief PM, Teresi JA, Eimicke JP, Shea S, Weinstock RS  2009  USA | Longitudinal data from randomized trial with physiological data and questionnaires.  Home telemedicine unit (web-enabled computer). | Diabetes type 2  N=1665 baseline; 1443 over time  No specific information regarding age; subjects were 55 years or older  2 year study duration  No information about research ethics |  | X |  |  | High |
| Umapathy H, Dickson C, Dobson F, Fransen M, Jones G, Hunter DJ  2015  Australia | Quasi-experimental design with questionnaires at baseline and after 12 months.  Publicly available web-based resource (website). | Hip and/or knee osteoarthritis  N=277 at baseline; 195 after 12 months  Mean age=61  12 month study duration  Research ethical approval |  | X |  |  | Medium |
| Whitten P, Bergman A, Meese MA, Bridwell K, Jule K  2009  USA | Mixed method: pre and post questionnaires and interviews.  Home telehealth service with remote monitoring. | Heart failure  N=50 (of which 35 were also in interviews)  Mean age=78  60 day study duration  No information about research ethics | X | X | X | X | Medium |
| Yu DX, Parmanto B, Dicianno BE, Pramana G  2015  USA | Lab tests with observations and questionnaires.  Mobile health system (applications). | Spina bifida  N=6  23-36 years old  Mean age=29  2 hour lab experiment  Research ethical approval |  |  | X | X | High |
| Zan S, Agboola S, Moore SA, Parks KA, Kvedar JC, Jethwani K  2015  USA | Single-arm prospective pilot study with questionnaires.  Mobile web-based telemonitoring system with IVR. | Heart failure  N=20  21-81 years old  Mean age=53  90 day study duration  Research ethical approval |  | X | X | X | High |
| Notes: RCT = Randomized controlled trial; I = intervention group; C = control group; IVR = interactive voice response; COPD = chronic obstructive pulmonary disease; CKD = chronic kidney disease.  ^1^Quality appraisal: low, medium, or high quality. | | | | | | | |
